# 

**Published:** 1911-07

**Authors:** 


### BOOKS RECEIVED

A Manual of Diseases of Infants and Children. By John Ruhrah, M.D., Clinical Professor of Diseases of Children, College of Physicians and Surgeons, Baltimore. Third revised edition. 12mo volume of 534 pages, fully illustrated. Philadelphia and London: W. B. Saunders Company. 1911. (Flexible leather, $2.50 net.)

Hospital Management. A Handbook for Hospital Trustees, Superintendents, Training School Principals and Physicians. By Charlotte A. Aikens, Author of "Hospital Training-School Methods and the Head Nurse." 12mo of 488 pages, illustrated. Philadelphia and London: W. B. Saunders Company. 1911. (Cloth, $3.00 net.)

Progressive Medicine, June, 1911. A Quarterly Digest of Advances, Discoveries and Improvements in the Medical and Surgical Sciences. Edited by Hobart Amory Hare, M.D., Professor of Therapeutics and Materia Medica in the Jefferson Medical College, Philadelphia, and Leighton F. Appleman, M.D., Instructor in Therapeutics in Jefferson Medical College. Philadelphia and New York: Lea & Febiger. (Per annum, paper bound, $6.00; cloth, $9.00.)

Joint Tuberculosis. By Leonard W. Ely, M.D., Consulting Orthopedist to the County Hospital; Attending Orthopedist to the Children's Hospital, Denver. 8mo, pp. 243. Illustrated. New York: William Wood & Co. 1911. (Cloth, $2.50.)

The Principles and Practice of Bandaging. By Gwilym G. Davis, M.D., Professor of Orthopedic Surgery, University of Pennsylvania. Third edition, revised. 12mo, pp. 128. Illustrated from original drawings by the author. Philadelphia: P. Blakiston's Son & Co. 1911. (Cloth, $1.00.)

Proceeding of the Canal Zone Medical Association. For the half-year, April, 1910, to September, 1910.

The Evolution of UrineAnalysis. By Burroughs Welcome & Co., London, Eng. 35 West 23d Street, New York.

### LITERARY NOTE

### INDEX FOR VOL. LXVI

### AUGUST, 1910, to JULY, 1911

| Page | | Page | |
|---|---|---|---|
| 1— 58 | August | 349—406 | February |
| 59—116 | September | 407—464 | March |
| 117—174 | October | 465—522 | April |
| 175—232 | November | 523—580 | May |
| 233—290 | December | 581—646 | June |
| 291—348 | January | 647—725 | July |

Page

ADDRESS to Medical Dept. U. of B. .................. 182
Angina Pectoris, nitrites and digitalis in .................. 434
Anglo-American memories ...... 75
Anniversary address, Drs. Clawson and Smith .............. 542
Army of the Potomac, three years with .................. 647
Arneth blood count, observations on .................. 204
Atophan in gout and rheumatism 681
BETA-EUCAIN .............. 264
Bethune, Charles W., tuberculosis of epididymis ......... 315
Bethune, Charles W., tuberculosis of epididymis ................ 315

**Books and Authors:**

Abrams: The blues.......... 579
Adam: Diseases of eye...... 518
Adami: Principles of pathology, Vol. I .............. 337
Adami: Systemic pathology.. 513
Allen: Vaccine therapy...... 229
Alger: Refraction and motility of eye................ 229
A.M.A.: New and non-official remedies, 1910, 1911.....57, 641
Ashurst: Fractures of humerus .................. 338
Ballenger: Diseases of nose, throat, ear .............. 574
Bandler: Vaginal celiotomy.. 520
Barnesby: Medical chaos and crime .................. 339
Barnhill: Modern otology... 517
Bassler: Diseases of stomach 516
Beck: Bismuth paste in suppurations ................ 514
Blakiston: Visiting list, 1911 338
Blech: Borderland surgery . 172
Bonney: Pulmonary tuberculosis .................. 54

Page

Boyd: State registration for nurses .................. 461
Bryant & Buck: American practice of surgery, Vol. VII .................. 108
Burrage: Gynecological diagnosis .................. 287
Cables: Diagnosis and treatment .................. 578
Cabot: Differential diagnosis. 519
Campbell: Surgical anatomy. 573
Cooper: Sexual disabilities in man .................. 340
Cotton: Dislocation and joint fractures .................. 229
Crandon: Surgical after-treatment .................. 53
Crossen: Diseases of women 515
Crothers: Inebriety ......... 722
Cushny: Pharmacology and therapeutics .............. 284
DeLorme: Manual of pharmacy .................. 460
Dock: Hookworm disease.... 340
Donahoe: Manual of nursing 459
Dugan: Electrotherapeutics.. 113
Egbert: Hygiene and sanitation .................. 227
Erlich: Anemia ............. 643
Faught: Laboratory diagnosis .................112, 228
Fein: Rhinology and laryngology .................. 460
Fenwick: Dyspepsia ........ 226
Fischer: Diseases of infancy. 280
Galbraith: Personal hygiene. 576
Gleason: Diseases of nose, etc 340
Gouraud: What Shall I Eat? 721
Gray: Anatomy ............. 228
Hare: Modern treatment..... 572
Hare: Progressive Medicine, June, '10, 56; Sept., '10, 225; Dec., '10, 462; March, '11... 641
Hart: Evolution and heredity 405
Hawes: Care of patient...... 460

Page
Head: Practical Medicine Series, 1910, Vols. 1 to 6, 333; Vols. 7 to 10 .............. 461
Henderson: Lessons on eye.. 462
Herschell: Duodenal ulcer .. 460
Herman: Difficult labor ..... 281
Hinsdale: Hydrotherapy .... 227
Hiss: Bacteriology ......... 457
Hollander: Brain disease.... 518
Hoxie: Symptomatic and regional therapeutics ....... 284
Hughes: Gynecology ........ 515
Hughes: Practice of medicine 642
Hutchinson: Food and dietetics ...................... 719
Ibershoff: Semicircular canals 113
Jellett: Midwifery .......... 169
Jolly: Atlas of gynecology... 578
Jordan: Bacteriology ....... 286
Kelly: Practice of medicine.. 404
Kerr: Care of children...... 462
King: Obstetrics ........... 282
Kisch: Sexual life of woman. 50
Kemp: Diseases of stomach and intestines ............. 51
Kolle: Plastic and cosmetic surgery .................. 579
Koplik: Diseases of infancy.. 110
Lea & Febiger: Visiting list, 1911 ...................... 283
Leftwich: Symtoms and diagnostic methods ......... 172
Lippincott: Dictionary ...... 459
Lippincott: International Clinics, 20th series, Vol. 2, 109; Vol. 3, 285; Vol. 4, 516; 21st series, Vol. 1......... 641
Longyear: Nephrocoloptosis. 224
Luckoff: Optic nerve and sinuses of nose ............. 111
McBride: Alcoholism ....... 520
McFarland: Pathology ...... 112
McFarland: Biology ........ 342
Manhattan Hospital: Nursing 228
Manquat: Therapeutics ..... 518
Mayo Clinic: Hospital Staff papers ................... 571
Morrow: Diagnostic and therapeutic technic ........ 640
Morris: Fourth era in surgery 278
Morse: Case histories in pediatrics ................... 577
Moynihan: Duodenal ulcer .. 49
Moynihan: Pathology of living ...................... 172
Mumford: Practice of surgery 336
Mummery: Diseases of colon 343
Musser and Kelly: Practical treatment, Vol. 1 ......... 575
Vol. II ........ 721
Noörden: Metabolism and nutrition ................... 170
O'Reilly: Physical diagnosis.. 580
Osler: Modern Medicine, Vol. 7 .......................... 55
Park: Pathogenic microörganisms ..................... 385
Piersol: Normal histology... 340
Price: Hygiene and public health .................... 342
Pyle: Personal hygiene...... 171
Reed: Diseases of stomach and intestines ............ 639
Ritchie: Primer of hygiene.. 462
Robinson: Never told tales.. 287
Rogers: Therapeutic action of light ..................... 229
Ross: Cell reproduction and cancer ................... 514
Rumpel: Cystoscopy in surgery ..................... 278
Sahli: Diagnostic methods... 577
Saunders: St. Mary's Hospital papers .................... 571
Schafer: Histology ......... 343
Schalek: Diseases of skin... 173
Schmidt: Function of intestines ..................... 111
deSchweinitz: Diseases of eye 52
Sheffield: Diseases of children ...................576, 642
Sisson: Veterinary anatomy.. 230
Slade: Diagnostic anatomy .. 227
Smith: What to eat and why 718
Squier: Manual of cystoscopy 716
Stedman: Dictionary ....... 718
Stelwagon: Diseases of skin. 336
Stewart: Physiology ........ 284
Stewart: Therapeutics and dose book ................ 113
Stitt: Bacteriology ......... 458
Stoney: Bacteriology and surgical technic ............. 342
Stoney: Nursing ............ 339
Sutton: Osteology and syndesmology ................ 459
Swan: Prescription writing.. 113
Tanner: Poisons ........515, 578
Taylor: Physicians' account book ..................... 342
Tousey: Medical electricity.. 52
Treves: Operative surgery... 230
Tuley: Obstetrical nursing.. 343
Tuttle: Principles of public health ................... 461
Warbasse: Animal experimentation ............... 171
Weeks: Diseases of eye...... 283
Whitby: Makers of man..... 573
Whitman: Orthopedic surgery ...................... 281
Wilcox: Treatment of disease 575

Page
Witthaus & Becker: Medical jurisprudence, Vol. IV .... 720
Wood: Ophthalmic operations, Vols. I and II...... 714
Wood: State board questions 463
Wood: Visiting list, 1911.... 282
Vecki: Sexual disease....... 405

**Reports:**

Johns Hopkins Hospital..... 113
Massachusetts General Hospital, Vol. 2 .............. 461

**Transactions:**

American Association Genito-urinary surgeons, 1908-'09.. 403
American Dermatological Association ................. 281
American Laryngological Socity, '09, 170; '10......... 461
Conference state and territorial health officers....... 341
Books received: 57, 173, 114, 231, 288, 344, 405, 463, 522, 580, 644, 723
Brovalol ........................ 264
Bulletin, Buffalo General Hospital ........................ 319, 685
CANCER, treatment of ....... 465
Cerebral compression ..... 648

**College and Hospital Notes:**

Barnard Free Skin and Cancer Hospital .............. 332
Buffalo General Hospital, 169, 277, 332, 403............... 619
Buffalo University, 107, 224, 169, 569 .................. 638
Children's Hospital, 454, 638, 711
Columbus Hospital, ......49, 713
Day Camp for Tuberculosis Patients ............. 709, 712
German Deaconess Hospital ...................... 454, 569
Homeopathic Hospital ...... 709
Lebanon Hospital .......... 332
New York University ....... 570
New York Skin and Cancer Hospital .................. 224
Riverside Hospital .......... 332
McGill University .......... 636
Pennsylvania University .... 277
Rockefeller Institute Hospital 223
State Library .............. 570
Sloane Maternity Hospital... 569
Tuberculosis Day Camp..... 635
Tuberculosis Hospital ....... 455
Wende, Ernest, Hospital.......................107, 455, 635
Connor, Leartus, conference of ophthalmologists .............. 14
Cycloplegia, differential diagnosis by ........................ 244

**Correspondence:** Page

Contagious diseases—Buffalo Health Department ....... 158
National Department of Health—Irving Fisher .... 35
Poliomyelitis—W. H. Frost. 395
Poliomyelitis epidemic—Robert W. Lovett ............ 97
DIGISTROPHAN ........... 267
Disease, N. Y. State Institute for malignant.. 581
prevention and cure of. 8
Diseases, moral and social, prevention of .......... 357
EGGS, bad, crusade against ................... 326, 399, 451
Ehrlich's "606" ................. 308
Enterocolitis, dysenteric, surgical treatment of .................. 311
Epilepsy ........................ 298
FINNIE, A. S., abuse of hospital and its cure ............ 247
Flies, crusade against ........... 164
GASTRIC diseases, "insane asylum" for .............. 187
Glaucoma, prevention of ........ 371
Goiter, exophthalmic, treatment of .......................... 588
Gonorrhea, chronic, in female.... 303
Gorgas, Colonel, work of ....... 324

Gould, George M., Differential diagnosis by cycloplegia .. 244
Epilepsy .. 298
Glaucoma, prevention of . 371
"Insane asylum" for gastric diseases . 187
Prevention and cure of disease.. 8
HAASE, Charles, serums and vaccines ................. 414
Hanley, L. G., abnormalities and complications of pregnant state.. 233
inguinal hernia.. 486
Hayd, Herman E., intussusception in infants ................ 291
Healthograms ...............40, 164
Heart nutritions ................ 407
Hegonon ...................... 266
Hernia, intraperitoneal ......... 301
Heyd, Charles G., observations on Arneth blood count........ 204
Hopkins, Henry R., the purins... 175

Page
Hormonol ..................... 432
Hormon in intestinal paralysis... 679
Hospital, abuse of ............. 247
bill, public ........... 41
Hospital delivery, maternity patients ........................ 532
ICHTHYOSIS ................ 129

**Illustrations:**

Infants, intussusception in ...... 291
Insane, detention ward for...... 103
Institute, N. Y. State .......... 581
Bethune, tuberculosis of epididymis .................... 316
Bethune, urethroscopic forceps ...................... 238
Frederick, Carlton C., portrait ..................... 633
Gould, prevention and cure of disease:
Figs. 1 and 2, drawing tables 10
Fig. 3, desk leaf raised.... 13
Heyd, Arneth blood count... 206
Long, sphymograms ........ 413
Potter, William Warren, portrait facing .............. 502
Potter, William Warren, portrait ...................... 648
Wallace, construction of vagina:
Fig. 1, rudimentary uteri.. 365
Fig. 2, sigmoid ........... 367
Fig. 3, sigmoid to form vagina ............... 369
Figs. 4 and 5, artificial vagina ............... 370
Wallace, intraperitoneal hernia:
Fig. 1. hernial sac........ 301
Fig. 2, duodeno-jejunal recess ............... 302
Wende, pellagra in Buffalo:
Plate 1, lesions of hands (insert) ........... 524
Plate 2, lesions of wrists (insert) ........... 528

**Items:**

Apollinaris ................. 346
Battle & Co., charts.....116, 290
Calox ...................... 346
Moet & Chandon, White Seal Champagne ............... 346

**LEADING Articles:**

American Association of Obstetricians and Gynecologists ..................... 159
Army, factors at head of.... 101
Bodine, Dr. J. M., testimonial dinner to ................ 272
Buffalo and the A. M. A. Meeting ................. 700

Page
Buffalo General Hospital, problems of .............. 38
Buffalo General Hospital, social service ............ 396
Cesarean section ........... 216
Cold storage ordinance...... 450
County Medical Society .... 559
Court, model ............... 220
Day Camp for Tuberculosis Patients .................. 703
Good pay to good men ..... 447
Health Officers, increased pay for .................. 703
Human life, value of ....... 443
Impure food, war on ....... 446
Instruments, case of lost..... 703
Janeway, Edward G. ........ 440
Jewett, Charles, in memoriam 322
Laboratory, State Cancer 397, 443
"Lest we Forget," U. of B. Alumni Banquet, April 27, 1900 ...................... 693
Institute, N. Y. State, Malignant Disease ............. 623
Massachusetts Metaphysical College and the Penal Code of the State of N. Y. ..... 697
Medicine of long ago ....... 561
Medical Examining Board, N. Y. State, changes in.... 702
Medical Society County of Erie ..................... 446
Medical Society State of N. Y., Tribute to William Warren Potter ........... 701
Nightingale, Florence ...... 98
Poliomyelitis and Dr. Frost. 321
Potter, William Warren, in memoriam, Alumni association, U. of B. .......... 562
Potter, William Warren—Lewis S. McMurtry ....... 502
Potter, William Warren, tribute, regents University State of N. Y. ............ 629
Potter, William Warren, tribute, State Board of Medical Examiners ............ 625
Price, Joseph, In Memoriam. 689
Reciprocity, New York and New Jersey ............... 162
Salvarsan, danger of ........ 441
Sanitary conference ........ 270
Smallpox situation .......... 703
The Sick as a political asset.. 699
Tuberculosis crusade ....... 219
University of Buffalo ....... 557
University of Buffalo, 1846-1911 ...................... 690
University day ............ 450

**Literary Notes:** Page
American Journal of Surgery 345
Breitenbach, M. J. Co.—Memorandum Book ...... 345
Davis, F. A. Co.—catalogue.. 232
Fellows Co.—Advertisement. 345
Harrower, Henry R., Physiologic Therapeutics ...... 289
Merck's Materia Medica, 1911 723
Saunders, W. B.,—Practical Treatment ............... 289
Schering & Glatz—Collargolum ..................... 645
Long, Eli H., heart nutrition.... 407
MACLEOD, J. A., and N. K., vaccines in surgical tuberculosis ...................... 16
McGuire, Edgar R., cerebral compression ..................... 658
Mason, Frederic S., therapeutic nihilism ..................... 306
Maternity patients, hospital delivery of ..................... 532
Medicine, early history in America ......................... 671
Medicine, future of ............ 188
Midwife, the .................. 477
Mills, public health news ....... 213
**Miscellany**
Abdominal supporter—H. W. Longyear ................. 725
Army medical corps examination ...................... 290
Battle & Co.—Charts........ 725
Henry Phipps Institute for Tuberculosis ............. 58
Suprarenal Situation—H. K. Mulford Co. .............. 724
U. S. civil service examinations .........116, 290, 348, 346
NASAL accessory sinuses, disease of .................. 75
Nurses, first training school for.. 39
**OBITUARY:**
Amsden, E. ................ 106
Babcock, Horace ........... 400
Beck, Carl B. ............... 707
Bickford, John W. ......... 454
Bull, A. T. ................. 106
Burke, J. J. A. .............. 106
Chester, Charles O. ........ 329
Cochrane, Royal E. ......... 274
Colvin, Darwin ............. 453
Dandridge, Nathaniel P. .... 274
Edwards, Landon B. ........ 328
Ely, William S. ............ 401
Goodfellow, George E. ..... 400
Greene, Charles A. ......... 454
Haller, John F. ............. 328
Hyde, James Nevins ....... 167
Janeway, Edward G. ....... 453
Page
Jewett, Charles ............ 105
June, Fred L. ............... 707
Kassabian, M. K. ........... 45
Kelly, A. O. J. .............. 511
Koyle, Frank H. ........... 453
Linville, Montgomery ...... 328
Macdonald, Willis G. ....... 400
Mason, John P. ............ 45
Moers, Emma W. ........... 707
Pohlman, Julius ............ 327
Potter, William Warren .... 509
Prince, Alpheus ............ 708
Ransom, Charles C. ........ 167
Searle, William S. .......... 328
Sherwood, Frank G. ........ 454
Strong, Thomas D. ......... 707
Van Peyma, W. ............ 107
Wiggin, F. W. ............. 274
Obstetrics and gynecology, progress in ..................1, 59, 117
PARK, Roswell, early history of medicine in America.... 671
Park, Roswell, treatment of cancer ......................... 465
Pellagra in Buffalo ............ 523
**Personal:**
Andrews, Herman D.; McGuire, Edgar R.; Seitz, George W.; Downing, Augustus S.; Wheelock, Charles F.; McKee, Thomas H. 705
Butler, Glentworth R.; Glosser, H. H. ................ 399
Diehl, Alfred E.: Lewis, F. Park; Park, Roswell; Jones, Allen A.; Fronczak, F. E.; Fuch, Professor E.; Gallivan, William F.; Snow, Irving M.; Hayd, Herman E.. 706
Ferguson, A. H., Putnam, J. W.; Stockton, Charles G.; Himmelsbach, G. A.; Stover, Charles; Stocker, G. B.. 105
B. ....................... 105
Fowler, Eva; Donk, Rose; Flood, Frances; Carrington, Henry O.; Wheeler, Justice Charles W. .............. 637
Fronczak, Francis E.; Park, Roswell; Cary, Charles; Sy, Albert P.; Rooth, H. C.; Creighton, S. S. .......... 104
Frye, Maud J.; Potter, William Warren; Grove, W. G. 632
Grove, Benjamin H. ........ 509
Hanley, L. G.; Bonifield, Charles L.; Haskell, Clayton K.; Vander Veer, Edgar A.; Cohen, Julius Y.; Heffron, John L. ............ 327

Page
Hendee, Lawrence; Crance, Charles T.; Wiley, Harvey W.; Woehnert, A. E.; Edmonds, Horace M.; Deaver, John B.; Ellis, George E... 452
Hiss, Philip H., Jr.; Butler, Glenlworth R. ............ 725
Hodge, William H.; May, William B.; Smith, Eugene A.; Rice, James F. ....... 274
Howell, S. Y.; Breuer, Max C.; Cary, Charles; Howe, Lucien; Fronczak, F. E.; Gaertner, William; Grant, John H.; Hitzel, G. A. .... 44
Howland, John D. .......... 453
Lewis, F. Park; Howe, William A.; Hayd, H. E.; Lynde, Uri C.; Pfaff; Orange G.; Hoyt, Robert E.; Palmer, Charles N. ... 436
Mason, Frederic S.; Porter, Eugene H.; Putnam, James W.; Allen, A. B.; Diehl, A. E.; Bodenbender, N. W.; McCormack, J. N. ........ 631
Norton, Chancellor Charles P.; Young, George B. ..... 707
Palmer, Charles N.; Hayd, H. E.; Fronczak, Francis E.; Simpson, Burton T. ...... 166
Rooth, H. C. ............... 273
Sheehan, Robert F.; Deaver, John B.; Schaefer, Arthur C.; Meahl, Charles S.; Smith, Winford H. ....... 563
Smith, Lee H.; Drake, John J.; O'Reilly, Charles; Mann, Badwin; Stocker, George B.; Paxton, R. A.; Hayd, H. E. ............. 508
Waterman, J. Hilton; Fronczak, F. E.; Kossel, A. .... 222
Wende, Grover W.; Miller, A. B. .................. 646
Physician and layman .......... 349
Physicians, letters to ........244, 371
Poliomyelitis, anterior .......... 165
Potter, William Warren, three years with army of Potomac... 647
Practitioner, the general ....... 198
Pregnancy, intrauterine ........ 595
Pregnant state, abnormalities of. 233
Prostatic hypertrophy, conservation in ...................... 135
Purins, the .................... 175
SALICYLATES, application of ........................ 677
Schroeter, Ludwik, diagnosis of intra-uterine pregnancy 595
Schroeter, Ludwik, hospital delivery of maternity patients 532
Sears, F. W., moral and social diseases ..................... 357
Smalley, George W., anglo-American memories ................ 76
Serums and vaccines ........... 414

**Society Meetings;**

American Academy of Medicine ..................275. 329
American Association of Clinical Research ......... 107
American Association of Obstetricians and Gynecologists ..................... 45
American Dermatological Association ................. 708
American Medical Association ...................... 568
American Medical Editors' Association ............... 708
American Public Health Association ...............47, 401
Buffalo Academy of Medicine, 223, 276, 330, 402, 456, 512, 567, ...................... 639
Buffalo Physical Education Society .................. 568
Health Officers Association of New York ............ 168
Homeopathic Medical Society 455
International Congress of Obstetrics and Gynecology... 48
International Congress of School Hygiene .......... 107
International Tuberculosis Congress ................. 167
Lackawanna Railroad Surgeons' Association ....... 708
Lake Keuka Medical and Surgical Association ......... 48
Medical Association of Central New York ........168, 276
Medical Society, County of Chemung ................ 331
Medical Society, County of Erie ..................331, 457
Medical Society, State of N. Y. ......329, 402, 455, 513, 565
Medical Society, State of N. Y. Eighth District Branch ...................... 168, 277
Medical Society, County of Ontario ................. 48
National Confederation of State Medical Examining and Licensing Boards ..... 329

Page
New York and New England Association of Railway Surgeons ....................222, 275
Ontario Medical Association. 708
Oregon State Board of Medical Examiners ........... 568
Rochester Academy of Medicine ......................
275, 455, 456, 402, 513, 568, 639
Southern Surgical and Gynecological Association ..276, 512

**Society Proceedings:**
American Medical Association ....................... 489
Association of American Medical Colleges ......... 498
Buffalo Academy of Medicine ...................... 604, 613
Buffalo Academy of Medicine 677
Medical Association of Central New York .........80, 140
Medical Society, County of Chemung ................. 391
Medical Society, County of Erie ......................
..26, 260, 374, 419, 487, 601, 613
Medical Society, County of Schuyler ................. 547
National Confederation of State Medical Examining and Licensing Boards 31, 90, 494
Spitting, anti, Chicago's plan.... 42
Stockton, Charles G., Address, anniversary Drs. Clawson and Smith .... 542
future of medicine, 188
Syphilis, treatment of ........... 436
TEETH, importance of examining ..................... 313
Therapeutic nihilism ............ 306
Tinker, Martin B., exophthalmic goiter ..... 588
surgical tuberculosis .... 68
Tuberculosis and vaccines....... 16
of epididymis .... 315
patients, day camp for ............. 165
surgical treatment of ............... 313
tubercle bacilli in feces ............ 258
Typhoid, anti, inoculation....... 437

**Topics of Public Interest** Page
Alcohol for hand disinfection 617
Census Bureau and vital statistics ..................... 269
Cold storage bill............ 682
Congress, School Hygiene... 682
Consumptives, public provision for .................. 268
Contagious Diseases Hospital ........................ 317
Cross, Double Red .......... 157
Examinations, civil service.. 215
Medical examining board, single, for Pennsylvania... 683
Midwife problem in America 504
Monarchs, queer medicines for ........................ 35
Paralysis, infantile ......... 211
Physicians Mutual Aid Association ................... 439
Poliomyelitis, acute epidemic 681
Poliomyelitis, microörganisms of ................... 500
Public health news ......... 213
Reciprocity treaty, New Jersey ....................... 32
Schools, open air for children 618
Schools, open air ........... 682
State Board of Regents..... 439
Tuberculosis association .... 212
Tuberculosis crusade ........ 210
Tuberculosis, fight against... 617
Tuberculosis war ........... 428
Tuberculosis war, women in. 93
UROTROPIN .......265, 431, 433

VACCINES in surgical tuberculosis ..................... 16
Vagina, construction of ......... 364
Van Peyma, P. W., the midwife. 477
WALLACE, W. L., construction of vagina .......... 364
Webster, Clarence J., obstetrics and gynecology ..........1, 59, 118
Wende, Grover W., physician and layman .... 349
pellagra in Buffalo .... 523
Wilson, Nelson W., prostatic hypertrophy .................. 135
Wood, General Leonard ........ 150
Wright, Adam H., the general practitioner ................... 198
Wright, Thew, address U. of B. . 182
YOUNG, A. A., ichthyosis.... 129

